# An Analysis of Differences in Successful Aging, Loneliness, and Depression According to Marital Status Among Older Golf Participants

**DOI:** 10.3390/bs16020266

**Published:** 2026-02-11

**Authors:** Hye Jin Yang, Ga-Young Kim, So-Jung Park, Chulhwan Choi, Chul-Ho Bum

**Affiliations:** 1 Department of Physical Education, Graduate School, Kyung Hee University, Seocheon-dong 1, Giheung-gu, Yongin-si 17104, Gyeonggi-do, Republic of Korea; y0108577@khu.ac.kr (H.J.Y.); buddysusan@gmail.com (G.-Y.K.); wannado14@khu.ac.kr (S.-J.P.); 2 Department of Physical Education, Gachon University, 1342 Seongnamdaero, Sujeong-gu, Seongnam-si 13120, Gyeonggi-do, Republic of Korea; 3 Department of Golf Industry, College of Physical Education, Kyung Hee University, Seocheon-dong 1, Giheung-gu, Yongin-si 17104, Gyeonggi-do, Republic of Korea

**Keywords:** older adults, widowhood, divorce, social isolation, emotional well-being, leisure, physical activity, golf participation, stress, quality of life

## Abstract

As the older adult population rapidly increases, society is entering an aged era, and attention to measures that promote healthy aging is growing. Golf, a widely practiced leisure sport, offers physical and psychological benefits for older adults. This study examined differences in successful aging, loneliness, and depressive mood according to marital status among older adults engaged in golf. A survey was conducted with 189 older adults. Data were analyzed using cross-tabulation, validity and reliability testing, multivariate analysis of variance, and post hoc tests. Statistically significant differences emerged across marital status groups. No significant differences were found in psychological aging. In physical and social aging, the married group showed more favorable outcomes than the divorced group, and in social aging, the bereaved group also scored higher than the divorced group. Emotional loneliness was greater among divorced and bereaved participants than among married ones, whereas social loneliness and depressive mood were highest in the divorced group. In sum, marital status was significantly associated with successful aging, loneliness, and depressive mood in older adults who play golf. Although golf participation was associated with more favorable psychological outcomes, divorced individuals remain particularly vulnerable in several domains, possibly owing to persistent social stigma.

## 1. Introduction

In contemporary society, advances in medical and industrial technologies have extended average life expectancy, resulting in rapid growth of the older adult population and the emergence of an aging society (Mathers et al., 2015). In Korea, adults aged 65 and over currently constitute 20% of the population in 2025, with projections indicating that this proportion will surpass 30% by 2040, attaining super-aged society status (Ministry of Data and Statistics, 2025). Such demographic shifts are concerning, as aging is frequently accompanied by physical decline, disease, and deterioration in mental health. Older adults often face stressful conditions including economic hardship, depressive mood, and loneliness, which may lead to serious psychological problems such as depression and suicide (Y. H. Choi & Kim, 2008; D. B. Kim & Park, 2010). Since 2021, the prevalence of depression and solitary death among older adults has steadily risen, particularly among those who are divorced or bereaved (S. Y. Kim, 2024; C. H. Lim, 2025). Consequently, population aging has become a major social concern, prompting increased interest in strategies that enable older adults to maintain healthy lives.

In this context, the concept of successful aging has gained prominence as a key determinant of quality of life in later years. Aging is often viewed negatively as a decline in sensory and functional abilities (Levy, 2009). However, it can also be seen as a dynamic process of change and development (Vaillant & Mukamal, 2001). Accumulated life experience can foster greater maturity in attitudes and values, thereby enhancing life satisfaction (Baltes & Baltes, 1993). Accordingly, research on successful aging has sought to reframe aging as a period of positive adaptation and growth. Building on this perspective, Rowe and Kahn (1998) introduced the framework of successful aging to promote meaningful and satisfying later life while countering negative stereotypes. Successful aging thus represents a critical construct for sustaining well-being amid the challenges of later life. Its core components encompass physical and mental health, family relationships, and active social engagement (An et al., 2011; Vaillant & Mukamal, 2001), all of which may be moderated by marital status.

In later life, the role of one’s spouse is particularly important. Spouses serve as both partners and friends who rely on each other throughout life (M.-H. Kim et al., 2004; Hoppmann & Smith, 2007). This relationship provides the psychological stability and social support necessary for successful aging (Moss & Moss, 2014). However, older couples may experience marital status changes, such as divorce or bereavement, over time or due to external circumstances. In bereavement, the most common experience among older couples, the passing of a beloved spouse can cause not only sadness but also profound suffering such as a deep sense of loss and loneliness for the surviving partner (Y.-J. Lee et al., 2007; Wilcox et al., 2003). Bisconti et al. (2004) found bereavement to be one of the most stressful events adults can experience, negatively affecting physical, mental, and social functioning and overall health. Loss of a spouse through divorce has also been shown to negatively impact psychological factors such as loneliness, isolation, and anxiety (M.-A. Lee, 2010). Thus, marital status can significantly impact successful aging and such psychological factors.

Older adults are particularly susceptible to loneliness. For older adults, loneliness involves feeling negative emotions when separated from the environments they belong to and have long experienced (Mullins et al., 1996), and experiencing greater loneliness when their current circumstances do not align with their expectations (Baumeister & Leary, 1995). Loneliness differs from solitude: loneliness is the distressing feeling that arises from involuntary social isolation, whereas solitude can result from voluntary separation (Hawkley & Cacioppo, 2010). Older adults are especially vulnerable to loneliness compared to other age groups due to factors such as reduced social capacity, physical aging, and changes in marital status like bereavement or divorce (Dahlberg et al., 2022). Deep and chronic loneliness in older adults significantly affects mental health (Ong et al., 2016) and can serve as a precursor to depression (Cacioppo et al., 2014; Luanaigh & Lawlor, 2008). Loneliness and depressive mood also increase the risk of diseases such as heart disease and stroke (Cacioppo et al., 2014), and can contribute to suicide among older adults, underscoring the importance of addressing these issues (Holt-Lunstad et al., 2015).

Depression encompasses a broader affective state than loneliness. It is characterized by persistent loss of interest, feelings of worthlessness, sadness, and an overall diminished emotional tone (Cai et al., 2023). Although depressive symptoms can occur at any age (Wilkinson et al., 2018), their prevalence is higher among older adults (Hasin et al., 2005). While mild depressive mood may not constitute a severe disorder, prolonged or intense episodes can progress to clinical depression, making it a critical variable in geriatric research (Jeon & Shin, 2011). Depression impairs physical and cognitive function and increases disease susceptibility (Rodda et al., 2011). Its severity in later life varies considerably with marital status (Alexopoulos, 2005). Following divorce, for instance, the absence of spousal care and exposure to social stigma can exacerbate withdrawal and depressive symptoms (Roh & Kang, 2012; Kawaguchi & Koike, 2016), underscoring the substantial influence of marital status on physical and psychological well-being.

However, divorce and bereavement do not necessarily lead only to negative outcomes. Many couples experience declining relationship satisfaction over long periods (Gottman & Notarius, 2002), and some who have endured prolonged marital conflict and stress report psychological liberation and greater autonomy after divorce, along with improved life satisfaction (H.-S. Lee, 2015). Carr (2004) observed that although the initial sense of loss after spousal bereavement is profound, self-esteem often recovers over time, and a positive self-perception can develop through independent living. These findings indicate that changes in marital status do not always affect older adults’ lives negatively and may vary depending on individual circumstances and environment. The impact of such changes can differ according to personal psychological resources, social relationships, and participation in leisure activities.

In recent years, the importance of leisure activities has been increasingly emphasized as a means of alleviating emotional and social difficulties among older adults. Among these activities, golf has attracted particular attention as a sport that promotes social interaction while supporting physical and mental health (Cann et al., 2005). As noted earlier, when older adults experience disruption of social ties due to marital status, golf is especially valuable because it is typically enjoyed in the company of others and positively influences interpersonal relationships (Y.-D. Choi et al., 2015). Moreover, golf involves low-intensity walking (Sell et al., 2008), enabling older adults to participate without excessive physical burden. Thus, participation in golf can serve as a meaningful leisure activity that reduces loneliness and depressive mood and promotes successful aging. Nevertheless, differences may still emerge depending on marital status. Previous research has shown that even when older adults engage in sports, satisfaction is higher when they participate together with a spouse (Kilbourne et al., 1990; Yang et al., 2024), suggesting that joint participation carries special significance; consequently, satisfaction may be lower following divorce or bereavement despite continued sports involvement.

Conversely, studies grounded in hedonic adaptation theory (Clark & Georgellis, 2013) suggest that regular physical activity can hasten recovery from the distress associated with bereavement (Hutchinson et al., 2003). The hedonic treadmill model also posits that subjective well-being generally returns to a baseline level following major life events (Brickman, 1971). These perspectives support the idea that participation in a sport such as golf can facilitate successful aging and alleviate loneliness and depressive mood even after bereavement or divorce. Furthermore, from the perspective of Activity Theory, which posits that human consciousness is formed through action (Wilson, 2008), it implies that an individual’s psychological state is not static but is continuously reshaped through interactions with the world—that is, through activity. Therefore, examining the effects of golf participation among older adults across different marital statuses is highly important. However, research exploring ways to adapt to the psychological and social changes resulting from shifts in marital status remains limited, and studies specifically addressing the role of golf in this adaptation process are particularly scarce.

Accordingly, the present study investigates differences in successful aging, loneliness, and depressive mood according to marital status among older adults who regularly participate in golf. By doing so, it aims to clarify the multifaceted influence of marital status on later-life well-being and to elucidate the moderating role of golf participation. The findings are expected to provide foundational evidence for designing targeted sports programs and informing policy initiatives for older adults.

## 2. Materials and Methods

### 2.1. Study Participants

This study targeted adults aged 65 years and older who were active golf participants. Marital status was classified into three categories: married, divorced, and bereaved. The age criterion of 65 years and older was adopted in accordance with standards established by the World Health Organization (2024). Participants were recruited through convenience sampling, a non-probability approach selected for its suitability to the study objectives. Snowball sampling was additionally employed, leveraging referrals from acquaintances and participants themselves, to secure an adequate sample size. Data collection was conducted online and in person over a 5-month period from April to August 2025. Although convenience and snowball sampling do not ensure full population representativeness, these methods were considered appropriate given the study’s specific focus on older adults who engage in golf and fall within the defined marital status groups. A total of 201 questionnaires were collected; after excluding 12 cases with incomplete or inconsistent responses, 189 valid responses were retained for analysis. Detailed sociodemographic characteristics of the participants are presented in Table 1.

### 2.2. Instruments

A structured self-report questionnaire consisting of 41 items was used. The instrument was organized into four sections: sociodemographic characteristics (6 items), successful aging (12 items), loneliness (8 items), and depressive mood (15 items). The successful aging scale, originally developed by D. Kim (2008), had been previously applied by Y. S. Kim (2018) in research on social support and successful aging among older learners. Loneliness was measured using the scale adapted by Vincenzi and Grabosky (1987), and depressive mood was assessed with the Geriatric Depression Scale–Short Form (Yesavage et al., 1982). All scales were reviewed, and minor adjustments were made to enhance conceptual clarity and contextual relevance for older adults who participate in golf. Responses were provided on a five-point Likert scale (1 = not at all, 5 = very much so), with higher scores indicating greater intensity of the construct measured.

### 2.3. Data Analysis

Data were analyzed using SPSS version 29.0. Descriptive statistics were computed to summarize participants’ demographic characteristics. Construct validity was assessed through exploratory factor analysis (EFA) using principal component extraction and varimax rotation. Factors were retained based on eigenvalues greater than 1.0, and items with factor loadings below 0.40 were considered for exclusion. Sampling adequacy was confirmed with the Kaiser–Meyer–Olkin (KMO) measure and Bartlett’s test of sphericity, while internal consistency was evaluated using Cronbach’s α coefficients. Pearson correlation coefficients were calculated to examine relationships among variables. Prior to conducting the multivariate analysis of variance (MANOVA), the assumption of homogeneity of variance-covariance matrices was tested using Box’s M test. Differences across marital status groups were tested with multivariate analysis of variance (MANOVA), followed by post hoc comparisons.

## 3. Results

### 3.1. Scale Validity and Reliability

The construct validity of the successful aging scale was assessed through EFA. The KMO measure was 0.878, and Bartlett’s test of sphericity was significant (*p* < 0.001), confirming suitability for factor analysis. Three subfactors emerged: psychological, physical, and social aging. Two items (Item 3 from physical aging and Item 4 from psychological aging) were excluded due to factor loadings below the acceptable threshold. Cronbach’s α values exceeded 0.700 for all subfactors (social aging: 0.796; physical aging: 0.740; psychological aging: 0.715), indicating acceptable internal consistency (Table 2).

For the loneliness scale, the KMO value was 0.879 and Bartlett’s test of sphericity was significant (*p* < 0.001), indicating appropriateness for factor analysis. Two distinct subfactors emerged: emotional loneliness and social loneliness. One item from the social loneliness factor (Item 4) and one from the emotional loneliness factor (Item 2) were excluded due to insufficient factor loadings. Cronbach’s α coefficients were 0.845 for social loneliness and 0.709 for emotional loneliness, both above the conventional 0.700 criterion (Table 3).

The depressive mood scale yielded a KMO value of 0.962 and a significant Bartlett’s test of sphericity (*p* = 0.001). EFA supported a unidimensional structure. The Cronbach’s α coefficient was 0.956, demonstrating excellent internal consistency (Table 4).

### 3.2. Correlation Analysis for Multicollinearity

Pearson correlation coefficients were calculated to assess relationships among the principal variables. Most correlations were statistically significant. Importantly, all coefficients remained below 0.80, indicating the absence of multicollinearity (Table 5).

### 3.3. MANOVA on Dependent Variables by Marital Status

To investigate the influence of marital status on successful aging, loneliness, and depressive mood among older adults who participate in golf, a MANOVA was conducted. Box’s M test confirmed the assumption of homogeneity of covariance matrices (Box’s M = 80.933, F = 1.834, *p* < 0.001). The multivariate test revealed a significant overall effect of marital status (Wilks’ Λ = 0.824, F = 3.076, *p* < 0.001, partial *η*^2^ = 0.093). Univariate follow-up tests indicated significant differences across groups in physical aging (F = 3.176, *p* = 0.044), social aging (F = 7.836, *p* < 0.001), emotional loneliness (F = 9.648, *p* < 0.001), social loneliness (F = 11.079, *p* < 0.001), and depressive mood (F = 3.431, *p* = 0.034). No significant difference was found in psychological aging (F = 1.061, *p* = 0.348). Detailed results are presented in Table 6. Specific group differences identified through post hoc comparisons are described in the following section.

Since the study examined differences among three marital status groups of older adults who participated in golf, post hoc tests were conducted to identify specific between-group variations. No significant group differences were found for psychological aging. For physical aging, the married group showed a higher mean score than the divorced group, whereas no significant differences emerged between the married and bereaved groups or between the divorced and bereaved groups. With regard to social aging, both the married and bereaved participants obtained higher mean scores than the divorced participants. Regarding emotional loneliness, the married group reported lower levels than the divorced and bereaved groups, with no significant difference between the latter two. For social loneliness, the married group exhibited the lowest mean score, followed by the bereaved group, which in turn had a lower score than the divorced group. In depressive mood, the married group recorded a lower mean score than the divorced group, while no significant differences were observed between the married and bereaved groups or between the bereaved and divorced groups. Detailed post hoc results and mean values for the dependent variables across the three groups are presented in Table 7 and Table 8.

## 4. Discussion

This study examined differences in successful aging, loneliness, and depressive mood among married, divorced, and bereaved older adults who regularly participate in golf. Accordingly, the findings are discussed with a focus on this specific group rather than the broader older adult population.

### 4.1. Successful Aging

No significant differences in psychological aging were observed across the three marital status groups. Older adults face a high risk of negative psychological states like alienation and depression (Hur & Yoo, 2002; Blazer, 2003), especially after spousal loss. However, the lack of group differences in this study suggests that regular golf participation may mitigate these risks. Engagement in social leisure sports fosters interaction and emotional bonding, thereby enhancing psychological stability (Lera-López et al., 2017). Consistent results have been reported among older adults living alone (Cho, 2014) and bereaved individuals (Wicker & Orlowski, 2021), where sports participation was associated with improved subjective well-being and reduced depression and stress.

Physical aging scores were significantly higher among married participants than among divorced participants. Age-related decline in physical function (Guo et al., 2022) renders spousal support increasingly important (Syse et al., 2022). Married individuals often benefit from mutual monitoring and encouragement of healthy behaviors (Gouin & Dymarski, 2024) as well as a higher likelihood of sustained sports engagement (Scheerder et al., 2006). Golf, as a moderate-intensity activity suitable for older adults (Kolt et al., 2004), may amplify these advantages when practiced with a spouse. In contrast, divorced older adults frequently face health disadvantages linked to the loss of spousal care (Lorenz et al., 2006; Lillard & Waite, 1995). Furthermore, previous studies (Joung et al., 1998; Bloom et al., 1978) also suggest a bidirectional relationship, whereby poorer health may elevate divorce risk.

No significant difference in physical aging emerged between the married and bereaved groups. This pattern may reflect the stronger social stigma attached to divorce in South Korea (H.-S. Lee, 2015; Lillard & Waite, 1995), which generates chronic stress and adversely affects health. Consistent evidence (Joung et al., 1998; Ding et al., 2021) indicates that divorced older adults experience poorer physical health and higher mortality rates than their bereaved counterparts.

Social aging was perceived more positively by both married and bereaved participants than by divorced participants. Leisure activities, particularly those involving regular social contact, are known to sustain relationships and promote successful aging (Menec, 2003). Golf, in particular, fosters a strong sense of belonging among older players (Stenner et al., 2019). Playing with a spouse or regular companions naturally strengthens interpersonal bonds (Hultsman, 2012; Song et al., 2020). Bereaved participants showed favorable scores, likely due to increased social support and empathy after their loss. Additionally, their active efforts to rebuild social networks may have contributed to these results (Awaliah et al., 2023; Steeves & Kahn, 2005; Holtslander et al., 2011). Divorced individuals, however, often encounter persistent stigma and subsequent withdrawal from social interactions, resulting in less favorable perceptions of social aging (Umberson & Karas Montez, 2010).

### 4.2. Loneliness

Married participants reported significantly lower emotional loneliness than both divorced and bereaved participants. The spouse serves as a primary provider of emotional support that is associated with lower levels of loneliness in later life (G. R. Lee & Ishii-Kuntz, 1987). Although golf participation contributes to reduced loneliness among older adults (Smale et al., 2022), the absence of a spouse appears to outweigh these benefits at the emotional level for divorced and bereaved individuals (Chappell & Badger, 1989). Spousal loss renders older adults especially vulnerable to emotional loneliness (Steeves & Kahn, 2005), consistent with Kiecolt-Glaser and Wilson (2017) findings of underscoring the unique protective role of marital partnership.

Social loneliness also varied systematically by marital status: lowest among married participants, intermediate among the bereaved, and highest among the divorced. These results align with evidence that spousal presence is associated with lower levels of loneliness in later life (Zisook & Shuchter, 1991). Group-based sports such as golf repeatedly reinforce social connections and reduce isolation (Suragarn et al., 2021; Eime et al., 2013; World Health Organization, 2020). Notably, bereaved participants experienced less social loneliness than divorced participants. Although loneliness often peaks during the first year following bereavement (Freak-Poli et al., 2022), surviving spouses typically receive considerable support from family and friends (Bergman & Segel-Karpas, 2021). In contrast, divorced older adults frequently face social stigma, labeling (Umberson & Karas Montez, 2010), and greater difficulty establishing new harmonious relationships (Disney et al., 2012; Malouff et al., 2010).

### 4.3. Depression

This study found that depressive mood was lower among married older adults than among divorced participants. The presence of a spouse provides emotional and instrumental support that is associated with lower levels of depressive mood (Musick & Bumpass, 2012). Previous studies have similarly shown that marriage acts as a psychological protective factor (Williams, 2003; Bulloch et al., 2017). Moreover, participation in leisure sports is related to lower levels of depression among older adults (Lawlor & Hopker, 2001; Netz et al., 2005). Golf, which combines outdoor activity, social interaction, and personal achievement (Summerall et al., 1999; Martin-García et al., 2025), may be particularly relevant in this regard (Upton, 2020).

This study also found no significant difference in depressive mood between married and bereaved participants. Although depressive mood typically rises immediately after bereavement, recovery often occurs through social support (Bonanno, 2004; Stroebe et al., 2007) and positive remembrance of the deceased (Carnelley et al., 2006). In contrast, divorced individuals may experience more persistent depression due to factors contributing to marital dissolution (Teachman, 2008). As dependence on a spouse increases with age, divorced older adults frequently show higher depressive mood than bereaved individuals (M.-A. Lee, 2010), consistent with the present findings.

### 4.4. Summary and Implications

This study found that married older adults participating in golf exhibited more favorable outcomes in successful aging, loneliness, and depressive mood than divorced or bereaved participants. The presence of a spouse appears to play a decisive role, and joint golf participation may further reinforce marital relationships. Fowers et al. (1996) found that relationship satisfaction often declines with age, making shared leisure activities particularly meaningful for maintaining emotional connection (Terr, 2000).

These findings indicate differences in mental health according to marital status among individuals who participate in golf. Married participants benefited from both spousal support and the activity itself, while bereaved individuals showed adaptive efforts to rebuild social ties through golf. Divorced participants, however, remained more vulnerable, likely due to social stigma. Thus, marital status remains an important factor related to quality of life in later years. Differences in quality of life are observed among individuals who participate in social leisure sports such as golf. Therefore, promoting sustained engagement in sports and leisure activities, while considering marital status differences, is recommended to enhance successful aging and mental health among older adults.

## 5. Conclusions and Limitations

This study examined differences in successful aging, loneliness, and depressive mood according to marital status (married, divorced, bereaved) among older adults who participate in golf. The main findings for this group are summarized below.

First, among the subfactors of successful aging, psychological aging showed no significant differences across marital status groups. However, physical aging was perceived more positively by married participants than by divorced participants, and social aging scores were higher in both the married and bereaved groups than in the divorced group. These results indicate that marital status significantly influences perceptions of physical and social aging.

Second, married participants reported lower levels of both emotional and social loneliness than divorced and bereaved participants. Notably, the married group exhibited the lowest social loneliness, while the bereaved group scored lower than the divorced group. These patterns underscore clear differences in the maintenance of social relationships depending on marital status.

Third, depressive mood was significantly lower in the married group than in the divorced group, whereas no significant differences emerged between the married and bereaved groups or between the bereaved and divorced groups. This suggests that depressive mood differs according to marital status and that differences in depressive mood are observed among individuals who regularly participate in golf.

Overall, the results demonstrate that marital status affects successful aging, loneliness, and depressive mood, with married older adults showing the most positive outcomes. The bereaved group also maintained social relationships, and differences in emotional stability were observed among bereaved individuals who participated in golf. In contrast, the divorced group exhibited greater vulnerability, likely due to social stigma and reduced social support.

Based on these findings, several recommendations are proposed. First, due to the cross-sectional nature of this study. In addition, the lack of baseline data prior to marital status transitions, there are limitations in clearly distinguishing the causes of the observed differences. Second, in the present study, gender differences were not analyzed separately. Since men and women may differ in how they cope with divorce and bereavement, future research should take gender differences into consideration. Third, the present study did not consider the time elapsed since spousal loss. Since levels of loneliness and depressive symptoms may vary depending on the duration following bereavement, future research should take the time since spousal loss into account.

In conclusion, this study confirmed that marital status and golf participation are important factors influencing successful aging and mental health among older adults. These findings have academic and practical significance as baseline data for developing welfare policies and sports programs that promote healthy and active aging.

## Figures and Tables

**Table 1:** Sociodemographic characteristics of older adults participating in golf by marital status.

		Married (G1)	Divorced (G2)	Bereaved (G3)
Older adults participating in golf		82 (43.4%)	50 (26.5%)	57 (30.2%)
Golf experience	Less than 1 year	32 (44.4%)	16 (22.2%)	24 (33.3%)
	1—less than 3 years	12 (44.4%)	5 (18.5%)	10 (33.3%)
	3—less than 5 years	9 (32.1%)	9 (32.1%)	10 (37.0%)
	5—less than 10 years	7 (22.6%)	13 (41.9%)	11 (35.5%)
	10—less than 20 years	22 (71.0%)	7 (22.6%)	2 (6.5%)
Frequency of golf practice	None	17 (54.8%)	6 (19.4%)	8 (25.8%)
	1–2 times a month	38 (40.4%)	28 (29.8%)	28 (29.8%)
	1–2 times a week	22 (42.3%)	12 (23.1%)	18 (34.6%)
	3–4 times a week	3 (30.0%)	4 (40.0%)	3 (30.0%)
	Almost every day	2 (100.0%)	0 (0.0%)	0 (0.0%)
Frequency of playing golf	None	12 (38.7%)	8 (25.8%)	11 (35.5%)
	1 time or less per month	47 (50.0%)	21 (22.3%)	26 (27.7%)
	2–4 times a month	22 (38.6%)	17 (29.8%)	18 (31.6%)
	5–7 times a month	1 (14.3%)	4 (57.1%)	2 (28.6%)
	Total	82 (43.4%)	50 (26.5%)	57 (30.2%)

Note. G1 = married; G2 = divorced; G3 = bereaved.

**Table 2:** Results of factor analysis for successful aging.

Items	1	2	3
SA-2: I have a close friend with whom I can share my true feelings.	0.792	0.060	0.301
SA-3: I regularly meet with my friends.	0.790	0.320	0.028
SA-1: I am recognized as a needed person in my social gatherings.	0.681	0.111	0.344
SA-4: I take an active interest in and participate in social affairs.	0.633	0.364	0.205
PA-1: I have little difficulty engaging in light physical activities.	0.204	0.830	0.101
PA-4: I feel physically healthier through exercise.	0.293	0.715	0.223
PA-2: I keep my appearance neat and clean.	0.095	0.676	0.397
PSA-1: I feel that my existence in this world has value.	0.158	0.305	0.787
PSA-3: I have plans for what I want to achieve in the remaining years of my life.	0.246	0.081	0.763
PSA-2: I feel that my past life has been meaningful.	0.268	0.307	0.600
Eigenvalue	4.563	1.059	0.920
Variance (%)	45.634	10.589	9.196
Cronbach’s alpha	0.796	0.740	0.715

Note. KMO = 0.878, χ^2^ = 671.287, df = 45, *p* < 0.001, SA = social aging, PA = physical aging, PSA = psychological aging.

**Table 3:** Results of factor analysis for loneliness.

Items	1	2
SL-1: I feel lonely	0.841	0.230
SL-3: I feel I have little to talk about with other people.	0.811	0.353
SL-2: I realize that I have not met or contacted anyone today.	0.796	0.347
EL-4: I do not feel that I am needed or important to others.	0.183	0.820
EL-3: When I talk about my problems with close acquaintances, they seem to feel burdened.	0.385	0.700
EL-1: I feel that I do not have any close friends.	0.334	0.682
Eigenvalue	3.523	0.693
Variance (%)	58.724	11.544
Cronbach’s alpha	0.845	0.709

Note. KMO = 0.879, χ^2^ = 456.592, df = 15, *p* < 0.001, SL = social loneliness, EL = emotional loneliness.

**Table 4:** Results of factor analysis for depression.

Items	1
Dep-13: I feel that I lack energy in my daily life.	0.873
Dep-5: I do not think I live a pleasant life.	0.870
Dep-7: I often feel that I am not happy.	0.862
Dep-8: I often feel lethargic.	0.854
Dep-12: I feel that my life is meaningless.	0.850
Dep-3: I feel that life is empty.	0.845
Dep-4: I often feel bored.	0.841
Dep-11: I think I lack vitality in my daily life.	0.839
Dep-1: I am generally dissatisfied with my life.	0.821
Dep-14: I feel that my current situation is hopeless.	0.807
Dep-2: I have recently lost interest or motivation in activities I used to enjoy.	0.751
Dep-6: I am afraid that something bad might happen.	0.739
Dep-15: I feel that I do not have any close friends.	0.695
Dep-9: I prefer staying at home to going out.	0.640
Dep-10: I feel that my memory is worse than that of others.	0.559
Eigenvalue	9.482
Variance (%)	63.213
Cronbach’s alpha	0.956

Note. KMO = 0.962, χ^2^ = 2268.932, df = 105, *p* < 0.001, Dep = depression.

**Table 5:** Results of the correlation analysis on dependent variables.

	1	2	3	4	5	6
Psychological aging	1					
Physical aging	0.568 **	1				
Social aging	0.568 **	0.547 **	1			
Emotional Loneliness	−0.393 **	−0.415 **	−0.437 **	1		
Social Loneliness	−0.432 **	−0.478 **	−0.446 **	0.669 **	1	
Depression	−0.605 **	−0.546 **	−0.444 **	0.428 **	0.683 **	1

Note. ** *p* < 0.01.

**Table 6:** Results of the MANOVA.

Variables	Subfactors	*df*	*F*	*p*	Partial *η*^2^	LSD
Successful Aging	Psychological aging	2	1.061	0.348	0.011	-
	Physical aging	2	3.176	0.044 *	0.033	a > b
	Social aging	2	7.836	<0.001 **	0.078	a,c > b
Loneliness	Emotional Loneliness	2	9.648	<0.001 **	0.094	a < b,c
	Social Loneliness	2	11.079	<0.001 **	0.106	a < b,c; c < b
Depression		2	3.431	0.034 *	0.036	a < b

Note. ** *p* < 0.01, * *p* < 0.05; a = married; b = divorced; c = bereaved; MANOVA: multivariate analysis of variance.

**Table 7:** Mean scores of the dependent variables by marital status.

Variables	Successful Aging			Loneliness		Depression
	1	2	3	4	5	6
Married	3.7602	4.0732	3.7500	2.2154	2.1626	2.2756
Divorced	3.6067	3.7733	3.2950	2.7467	2.8733	2.6227
Bereaved	3.7895	3.8596	3.6272	2.5029	2.5029	2.3930

Note. 1 = psychological aging, 2 = physical aging, 3 = social aging, 4 = emotional loneliness, 5 = social loneliness; 6 = depression.

**Table 8:** Results of the post hoc analyses from marital status.

Variables		Successful Aging			Loneliness		Depression
		1	2	3	4	5	6
Married	G2	0.222	0.019 *	<0.001 ***	<0.001 ***	<0.001 ***	0.010 *
	G3	0.808	0.082	0.271	0.016 *	0.021 *	0.358
Divorced	G1	0.222	0.019 *	<0.001 ***	<0.001 ***	<0.001 ***	0.010 *
	G3	0.179	0.530	0.009 **	0.068	0.025 *	0.111
Bereaved	G1	0.808	0.082	0.271	0.016 *	0.021 *	0.358
	G2	0.179	0.530	0.009 **	0.068	0.025 *	0.111

Note. *** *p* < 0.001, ** *p* < 0.01, * *p* < 0.05; G1 = married; G2 = divorced; G3 = bereaved; 1 = psychological aging; 2 = physical aging; 3 = social aging; 4 = emotional loneliness; 5 = social loneliness; 6 = depression.

## Data Availability

The data presented in this study are available on request from the corresponding author due to restrictions based on participant confidentiality.

## Abbreviations

The following abbreviations are used in this manuscript:

CC	Creative Commons
EFA	Exploratory factor analysis
MANOVA	Multivariate analysis of variance
